# Genome-wide association study of early-onset bipolar I disorder in the Han Taiwanese population

**DOI:** 10.1038/s41398-021-01407-6

**Published:** 2021-05-20

**Authors:** Lawrence Shih-Hsin Wu, Ming-Chyi Huang, Cathy Shen-Jang Fann, Hsien-Yuan Lane, Chian-Jue Kuo, Wei-Che Chiu, Pui-Yan Kwok, Andrew Tai-Ann Cheng

**Affiliations:** 1 Graduate Institute of Biomedical Sciences, China Medical University, Taichung, Taiwan; 2 Department of Psychiatry, Taipei City Psychiatric Center, Taipei City Hospital, Taipei, Taiwan; 3 Department of Psychiatry, School of Medicine, College of Medicine, Taipei Medical University, Taipei, Taiwan; 4 Institute of Biomedical Sciences, Academia Sinica, Taipei, Taiwan; 5 Department of Psychiatry, China Medical University Hospital, Taichung, Taiwan; 6 Department of Psychiatry, Cathay General Hospital, Taipei, 10630 Taiwan; 7 School of Medicine, Fu Jen Catholic University, Taipei, 24205 Taiwan

**Keywords:** Clinical genetics, Bipolar disorder

## Abstract

The search for susceptibility genes underlying the heterogeneous bipolar disorder has been inconclusive, often with irreproducible results. There is a hope that narrowing the phenotypes will increase the power of genetic analysis. Early-onset bipolar disorder is thought to be a genetically homogeneous subtype with greater symptom severity. We conducted a genome-wide association study (GWAS) for this subtype in bipolar I (BPI) disorder. Study participants included 1779 patients of Han Chinese descent with BPI disorder recruited by the Taiwan Bipolar Consortium. We conducted phenotype assessment using the Chinese version of the Schedules for Clinical Assessment in Neuropsychiatry and prepared a life chart with graphic depiction of lifetime clinical course for each of the BPI patient recruited. The assessment of onset age was based on this life chart with early onset defined as ≤20 years of age. We performed GWAS in a discovery group of 516 early-onset and 790 non-early-onset BPI patients, followed by a replication study in an independent group of 153 early-onset and 320 non-early-onset BPI patients and a meta-analysis with these two groups. The SNP rs11127876, located in the intron of *CADM2*, showed association with early-onset BPI in the discovery cohort (*P* = 7.04 × 10^−8^) and in the test of replication (*P* = 0.0354). After meta-analysis, this SNP was demonstrated to be a new genetic locus in *CADM2* gene associated with early-onset BPI disorder (*P* = 5.19 × 10^−8^).

## Introduction

Bipolar disorder (BPD) is a severe, chronic, and disabling mental illness characterized by recurrent episodes of hypomania or mania and depression^1,2^. It is a clinically defined nosological entity with multifactorial but poorly understood etiologic mechanisms. The evidence from twin, family, and adoption studies provide compelling evidence for a strong genetic predisposition to BPD^3^ with heritability estimated to be as high as ≥80%^3–5^.

Given BPD is a heterogeneous disease with substantial phenotypic and genetic complexities^6^, the identification for BPD risk loci has proven to be difficult. Some researchers have proposed that dissecting BPD into clinical subgroups with distinct sub-phenotypes may result in genetically homogeneous cohorts to facilitate the mapping of BPD susceptibility genes^6^. Among the sub-phenotypes, early-onset BPD is of particular interest as several independent cohort studies have demonstrated their existence^7–11^.

Comparing to the non-early-onset BPD, the early-onset subtype is associated with a more severe form of clinical manifestations characterized by frequent psychotic features, more mixed episodes, greater psychiatric co-morbidity such as drug and alcohol abuse and anxiety disorders^12,13^, higher risk of suicide attempt^14^, worse cognitive performance^15^, and poorer response to prophylactic lithium treatment^13,16–20^. In addition, the pattern of disease inheritance seems to differ between early‐ and late‐onset BPD families^21,22^, with the former involving greater heritability^23^. These observations indicate that early-onset BPD may be a genetically homogenous subset and thus could be used for genetic study to identify its susceptibility genes.

A number of BPD genes identified by genome-wide association study (GWAS) have been widely replicated and intensively studied (e.g., *CACAN1C* and *ANK3*)^24^, but these studies did not include early-onset BPD. Over the past two decades, a host of studies have investigated genetic loci responsible for early-onset BPD through linkage-analyses, candidate–gene association, analyses of copy number variants (CNVs), and GWAS, but findings are inconclusive. Candidate–gene association studies have identified a number of genes potentially associated with early-onset BPD, including glycogen synthase kinase 3-β gene^25^, circadian clock gene Per 3^26^, serotonin transporter gene^27^, brain-derived neurotrophic factor gene^28^, and gene coding synaptosomal-associated protein SNAP25^29^. However, very few positive findings of these studies have been replicated independently. Findings from linkage studies suggested chromosomal regions harboring the susceptibility genes at 3p14 and 21q22, plus the loci at 18p11, 6q25, 9q34 and 20q11 with nominal significance^21,30,31^. Studies of CNVs in early-onset BPD were based on relatively small effect sizes and were irreproducible, suggesting that CNVs are unlikely the major source of liability^32–35^. Finally, GWAS failed to find any risk variant with genome-wide statistical significance in Caucasian populations, despite some variants showed suggestive significance^36^.

In previous genetic studies, the definition of early-onset in BPD typically ranged from 15 to 25 years of age. These association studies were largely conducted with small sample size and were underpowered (<80%)^36,37^. Most of them (including GWAS) compared early-onset BPD vs. healthy control. Such a case–control design is more likely to identify susceptibility gene(s) for BPD per se, but not for the early-onset subtype. The optimal strategy to identify gene(s) for the early-onset BPD is to include the non-early-onset BPD group for comparison. Different definitions for early onset of BPI have been proposed in previous work. In this paper, we reported findings from a GWAS with high-density SNP chips on early-onset, defined as ≤20 years of age, BPI patients of Han Taiwanese descent.

## Materials and methods

### Study participants

Study participants included a total of 1779 unrelated Han Taiwanese BPI patients recruited from psychiatric departments of general hospitals and psychiatric institutions in the Taiwan Bipolar Consortium^38,39^.

### Phenotype definition and assessment

The clinical phenotype assessment of manic and depressive episodes was performed by well-trained psychiatric nurses and psychiatrists using a cross-culturally validated and reliable Chinese version of the Schedules for Clinical Assessment in Neuropsychiatry^40^, supplemented by available medical records. All of them were diagnosed according to the DSM-IV criteria for BPI disorder with recurrent episodes of mania with or without depressive episode(s).

The assessment of onset age was based on a life chart prepared with graphic depiction of lifetime clinical course for each of the BPI patient recruited^41^. This life chart included largely all the mood episodes with date of onset (year and month), duration, and severity (including the extent of functional disability, hospitalization, and the presence of psychotic features). The construction of this life chart was based on integrated information gathered from direct interview with patients and their family members, interviews with in-charge psychiatrists, and a thorough medical chart review.

Different definitions for early onset of BPI have been proposed in previous work. Our selection of 20 as the age threshold was based on a systematic review for pediatric BPD^42^. The age at onset was defined by the first mood episode (depressive, manic, hypomanic, or mixed). Of all patients, 1306 (516 with onset age ≤20 and 790 > 20) with genotyping data available were included in the discovery group for GWAS and the rest 473 (153 with onset age ≤20 and 320 > 20) without genotyping data were included in the replication group.

### Genotyping

Genotyping was performed using the Illumina HumanOmni1-Quad BeadChip (*N* = 936) and the HumanOmni2.5-Quad BeadChip (*N* = 575) by Chun-Tai Co. (Taipei, Taiwan). For this study, we integrated the two genome-wide SNP data sets through imputation with 1000 Genomes. To bridge the two sets, we also genotyped 82 of the first 936 participants using the HumanOmni2.5-Quad BeadChip. The two gene chips shared about 750 K common SNPs; thus, we can check the genotyping consistency of the two sets. Genotype calling for the two data sets was determined by BeadStudio (Illumina) using default parameters. The genotype imputation method, IMPUTE2^43^, was performed under default setting to estimate the genotypes of SNPs not on array. In the imputation process, reference haplotypes was curated from 1000 Genomes Project Phase III^44^. To improve the efficiency, we performed a whole genome imputation in every 5 Mb chunk, respectively. ANNOVAR^45^ was used to annotate the functional consequences of single-nucleotide variants found in our data set. The imputation data set included genotyping information from 1429 (936 + 575 – 82) patients and 1306 of them with clear onset-age data were included in the discovery group for GWAS.

The subsequent quality control of the genotype data was implemented for each data set to exclude the following SNPs and individuals before the imputation: (1) individuals with a call rate <98%; (2) *P* < 1.0 × 10^−4^ for Hardy-Weinberg violation; (3) SNPs with MAF < 5%; (4) samples with first-degree cryptic relationships; and (5) samples that were potentially contaminated.

We used the Agena MassARRAY platform with iPLEX chemistry (Agena, San Diego, CA) for replication and confirmed the genotyping result from SNP arrays. The Spectro-CHIPs were analyzed using the MassARRAY Analyzer 4, and the results were analyzed using clustering analysis with the TYPER 4.0 software. The allele-specific diagnostic products had a unique molecular weight that were identified using matrix-assisted laser desorption ionization time-of-flight mass spectrometry.

### Statistical analysis

A principal component analysis (PCA) with 1306 patients in discovery group based on the genome-wide IBS (identical by state) pairwise distances was performed using PLINK v. 1.9 (https://www.cog-genomics.org/plink2)^46^. GWAS was carried out for the discovery group, by comparing allele frequencies between early-onset (≤20) and non-early-onset (>20) BPI patients. The threshold *P* value was set at 1.05 × 10^−8^ after a Bonferroni correction for the number of SNPs (4,750,978, including imputed SNPs). Because few SNPs reached the significance after Bonferroni correction, top SNPs with *P* values less than 10^−7^ were also considered for further examinations. Quantile–quantile (Q–Q) plots were then used to examine the *P* value distributions. The calculation of GWAS and Q–Q plots was performed using PLINK v. 1.9^46^. Top SNPs identified in the GWAS were replicated with 473 patients in the replication group. A meta-analysis was then performed by SAS 9.4 (SAS Institute Inc., Cary, NC, USA), using its existing macros. We adopted the macro as “*x* = –2 × (log(GWAS *P* value) + log(replication *P* value)), metaP = 1 – CDF(‘CHISQUARE’,*x*,4)” to calculate the *P* value, where CDF represents cumulative distribution function. We ran meta-analysis using SAS existing macro based on *P* values from GWAS and replication. The meta-analysis was also performed by PLINK v. 1.9^46^ based on the odds ratio (OR), standard error of OR, and *P* values.

## Results

Demographic and clinical characteristics of the patients in the discovery (*n* = 1306) and the replication (*n* = 473) groups are shown in Table 1. Female preponderance was observed in both groups and in all early- and non-early-onset subgroups, but the difference was not statistically significant in all of them. Rates of psychotic features and suicide attempt were higher in early-onset subgroups than in their non-early-onset counterparts in both discovery and replication groups (90.8% versus 84.2% and 90.2% versus 80.9% for psychotic features; 51.1% versus 43.7% and 41.8% versus 34.3% for suicide attempt, respectively). A higher proportion of the first mood episode was found to be depression in the early-onset subgroup in both discovery and replication groups (45.2% and 39.9%, respectively). The difference was statistically significant in the discovery group (χ^2^ = 7.9117, *P* = 0.005).

**Table 1:** Demographic and clinical characteristics of study patients with bipolar I (BPI) disorder^a^.

	Discovery group			Replication group		
	Onset ≤ 20 (*N* = 516)	Onset > 20 (*N* = 790)	*P* value	Onset ≤ 20 (*N* = 153)	Onset > 20 (*N* = 320)	*P* value
*Sex*			0.843			0.938
Male	229 (44.4%)	355 (44.9%)		74 (48.4%)	156 (48.7%)	
Female	287 (55.6%)	435 (55.1%)		79 (51.6%)	164 (51.3%)	
*Age at onset*	17.02 ± 2.54	31.83 ± 9.29	<0.001	17.65 ± 1.99	32.05 ± 9.03	<0.001
*Age at recruitment*	37.56 ± 11.79	41.84 ± 11.63	<0.001	37.56 ± 12.52	47.23 ± 12.35	<0.001
*With psychotic features (%)*	465 (90.8%)	665 (84.2%)	0.002	138 (90.2%)	259 (80.9%)	0.010
*With suicide attempt*	264 (51.1%)	345 (43.7%)	0.008	64 (41.8%)	110 (34.3%)	0.116
*First mood episode*			0.005			0.685
Mania	283 (54.8%)	495 (62.7%)		92 (60.1%)	205 (64.1%)	
Depression	233 (45.2%)	295 (37.3%)		61 (39.9%)	115 (35.9%)	

^a^Including early-onset (≤20) and non-early-onset (>20) patients in both discovery and replication groups.

### Association analysis

**Fig. 1:**
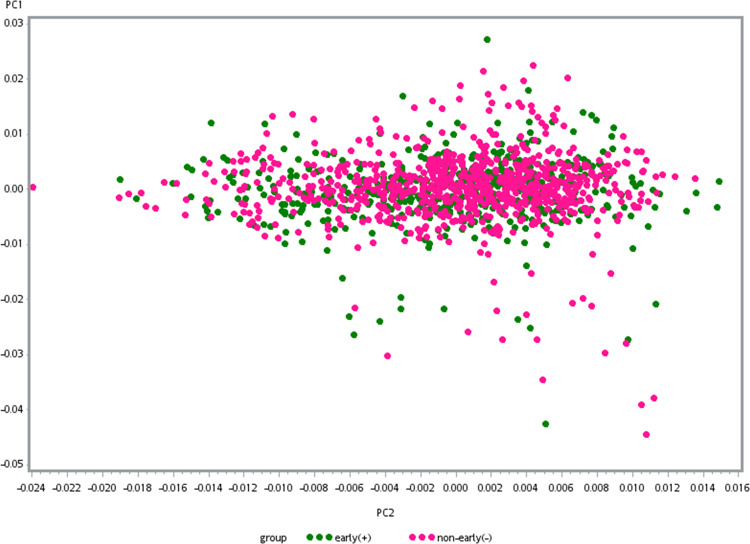
The principal component analysis plot of the 1306 GWAS samples. The Y and X axes are the first and second dimensions from principal component analysis based on the genome-wide IBS pairwise distances among the 1306 GWAS subjects. Green crosses represented early-onset and pink non-early-onset BPI. The two axes correspond to a reduced representation of 10,000 randomly selected SNPs into two dimensions. No clustering pattern was found, indicating that neither substantial population stratification nor cryptic relationship among the 1306 subjects was found.

**Fig. 2:**
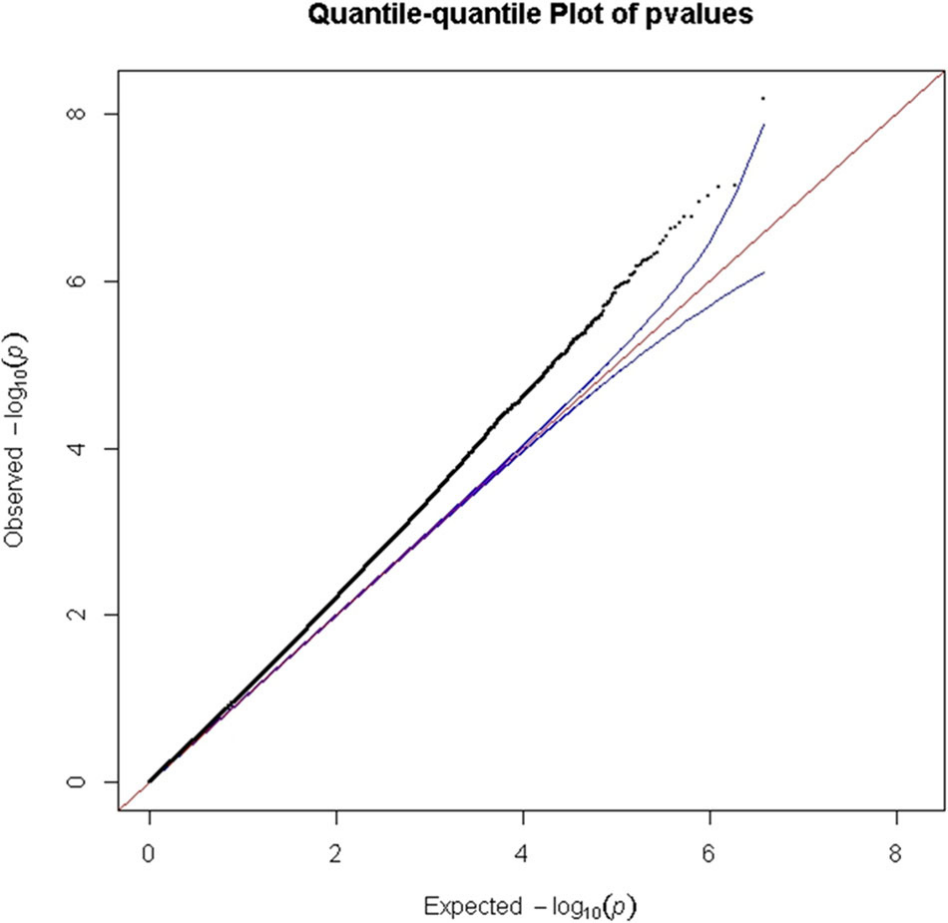
Q–Q plot of the Fisher’s test for allelic model *P* values in the discovery group of BPI disorder (*N* = 1306). The upper and lower boundaries of the 95% confidence bands are represented by the blue lines.

**Fig. 3:**
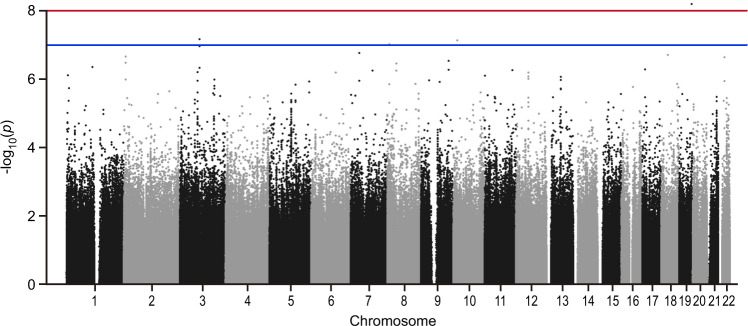
Genome-wide association between single-nucleotide polymorphisms (SNPs) and early-onset BPI disorder in the discovery group. The negative log of the *P* value for the association for allelic model was plotted against the chromosomal location across the genome. The upper and lower horizontal lines indicate the genome-wide significance level of 1.05 × 10^−8^ and 10^−7^, respectively.

Genotyping consistency of the common SNPs in the two sets (HumanOmni1-Quad BeadChip and HumanOmni2.5-Quad BeadChip) was checked in 82 sample and the average genotyping consistency of >99.98% was found. The PCA was used to present the genotyping consistency (Supplementary Fig. 1). PCA did not find substantial population stratification and cryptic relationship among the 1306 GWAS subjects (Fig. 1). The PCA was also performed to check whether there is a sampling bias between the two chips. No stratification and cryptic relationship was found between subjects with each of them (Supplementary Fig. 2). Figure 2 shows the Q–Q plot from results of the GWAS for early-onset BPI and the lambda value was 1.067. Associations between individual SNPs and early onset in 1306 patients with BPI calculated by χ^2^ (1df) for basic allelic test was plotted against the chromosomal location across the genome (Fig. 3). The horizontal line indicates the genome-wide significance level of *P* = 1.05 × 10^−8^, achieved by one SNP located on upstream of *MIR522*. In addition, three SNPs reached nominal significance at 1.05 × 10^−8^ < *P* < 10^−7^. They were located in the intron of *CADM2* (cell adhesion molecule 2) and *CUBN* (cubilin), and in intergenic region between *MFHAS1* (malignant fibrous histiocytoma amplified sequence 1) and *ERI1* (exoribonuclease 1) (Table 2), respectively.

**Table 2:** Distribution of allele frequency and *P* value of top four SNPs significantly associated with early-onset BPI disorder (≤20 years of age) in discovery and replication groups.

SNP ID	CHR	BP^a^	Gene/Feature	Allele	Group	Minor allele frequency (%)		*P*	OR (95% CI)	*P*_meta(SAS)/_ *P*_meta(PLINK)_
						≤20	>20			
rs75928006	19	54254388	*MIR522*/upstream	C/T	Discovery	9.4	3.9	6.45 × 10^−9^	2.58 (1.86, 3.60)	9.23 × 10^–8^/ 8.14 × 10^−5^
					Replication	15.7	16.7	0.7084	0.93 (0.64, 1.34)	
rs11127876	3	85124697	*CADM2*/intronic	C/T	Discovery	9.3	4.1	7.04 × 10^−8^	2.39 (1.73, 3.31)	5.19 × 10^−8^/ 1.26 × 10^−7^
					Replication	21.2	15.6	0.0354	1.46 (1.03, 2.06)	
rs7914868	10	16973722	*CUBN*/intronic	C/G	Discovery	12.4	6.3	7.59 × 10^−8^	2.10 (1.59, 2.76)	7.00 × 10^−7^/ 8.31 × 10^−6^
					Replication	24.5	22.5	0.5102	1.12 (0.81, 1.54)	
rs10106565	8	8855032	*MFHAS1-ERI1*/intergenic	C/T	Discovery	9.8	4.5	9.70 × 10^−8^	2.31 (1.68, 3.16)	1.33 × 10^−7^/ 4.38 × 10^−5^
					Replication	18.6	17.8	0.7866	1.06 (0.74, 1.50)	

*P* values, ORs, and CIs were calculated based on the allele model.

*OR* odds ratio, *CI* confidence interval, *P*_*meta (SAS)*_, *P*_*meta (PLINK)*_ *P* value of meta-analysis generated by SAS and PLINK v. 1.9 program.

^a^Location was cited according to GRCh37.p13.

These four top SNPs were then genotyped in the replication group. The SNP rs11127876, located in the intron of *CADM2*, showed nominal association (*P* = 0.0354, not resist to correction for multiple testing) (Table 2). The association in GWAS (discovery group) of SNPs in *CADM2* genomic region was further checked and some SNPs near rs11127876 also showed nominal association (Supplementary Fig. 3). We finally performed meta-analysis for both discovery and replication groups. The SNP rs11127876 showed the top association in both approaches (*P* = 5.19 × 10^−8^ using SAS; *P* = 1.26 × 10^−7^ using PLINK) close to genome-wide significance.

## Discussion

In this paper, we have reported one of the largest GWASs to investigate genetic susceptibility to early-onset BPI with the first mood episode occurring ≤20 years of age. We employed standardized psychiatric interview and constructed a life chart with detailed clinical history to ensure the accuracy and homogeneity of phenotype for genotyping. Our GWAS with high-density SNP chips identified the SNP rs11127876 in *CADM2* gene to be associated with early-onset BPI in both discovery and replication groups, and meta-analysis for the association was close to genome-wide significance (*P* = 5.19 × 10^−8^).

The gene *CADM2* on chromosome 3 encodes a synaptic cell adhesion molecule that is prominently expressed in neurons, and plays key roles in the development, differentiation, and maintaining synaptic circuitry of the central nervous system^47^. In previous GWASs, *CADM2* has been found to be associated with a number of mental health-related traits, including alcohol consumption^48^, cannabis use^49,50^, reduced anxiety, neuroticism and conscientiousness, and increased risk-taking behavior^51,52^. *CADM2* was also reported to be associated with executive functioning and processing speed^53^, general cognitive function^54^, and educational attainment^55^.

Though there have been no investigations examining the risk-taking phenotype in early-onset relative to non-early-onset BPD, Homes et al. showed that BPD patients with a past history of alcohol abuse or dependence had a higher risk-taking propensity^56^, suggesting a relationship between early-onset BPD and risk-taking propensity.

Of note, Morris et al. suggested that *CADM2* variants may not only link with psychological traits, but also influence metabolic-related traits, such as body mass index, blood pressure, and C-reactive protein^57^. In addition, they found that *CADM2* variants had genotype-specific effects on CADM2 expression levels in adult brain and adipose tissues. The finding highlights the potential pleiotropy of *CADM2* gene, i.e., the genetic variants may influence multiple traits, and shared biological mechanisms across brain and adipose tissues through the regulation of *CADM2* expression^57^. Given that the metabolic comorbidities are prevalent in patients with early-onset BPD^58^, it is conceivable that *CADM2* variants may influence both psychological (emotional processing) and physical (metabolic regulation) traits, further contributing to a more severe clinical subtype of BPD with accompanying risk of metabolic adversities. In addition, an association between risk-taking and obesity has also been implicated in previous research, which suggests that risk-takers tend to overlook health-related outcomes and are prone to aberrant reward circuitry predisposing them to poor dietary choices and excessive intake^57,59^.

Collectively, in line with the characteristics found to be associated with *CADM2* variants, it is likely that *CADM2* may exert an effect on the constellation of clinical features related to early-onset BPD with greater symptom severity (Table 1). Therefore, our findings suggest that *CADM2* genetic variants may have significant effects on a subtype of BPD with early-onset.

Two previous GWASs comparing early-onset BPD patients with healthy controls did not find any genetic variants reaching genome-wide significance^36,37^. Our study included a larger sample of early-onset BPI patients (*N* = 669) to conduct GWAS using high-density genotyping (4,750,978 SNPs generated by gene chips and imputation). The statistical power was calculated using Post-hoc Power Calculator (https://clincalc.com/stats/Power.aspx), combining the allelic frequencies of both discovery and replication groups. In this study of two independent samples of BPI with dichotomous endpoint, the power reached 99.4% and 18.2% under type I error (*α*) = 0.05 and = 5 × 10^−8^, respectively. Results of our study are also likely to be underpowered under the stringency setting of type I error. However, the frequency of risk allele T was higher in patients with onset ≤20 than in patients with onset >20 in both discovery and replication groups. We believe all these have provided strong evidence to confirm the association of this SNP with early-onset BPD.

In Table 2, the minor allele frequencies differ quite a bit between the discovery and replication cohorts. In the NCBI SNP database, minor allele frequency of rs11127876 is 0.08 (T) in Han Chinese in Beijing, close to our results and suggest that the different allele frequencies observed in Table 2 may mainly result from our sampling. The over-representative minor allele frequency in replication group may have come from random sampling or effects of hidden characters (phenotypes) of our patients recruited. Genetic variant of *CADM2* has been reported to be associated with behavioral and metabolic traits^57^, which were not assessed in this study. Though the minor allele frequencies of rs11127876 were different in discovery and replication groups, the same direction of ORs of rs11127876 minor allele supports the reliability of our findings.

The SNP rs75928006 located in the upstream of *MIR522* reached genome-wide significance in discovery group but failed to show statistical significance in replication group. *MIR522* promotes glioblastoma cell proliferation^60^, but there was no evidence to suggest its association with any psychiatric disorders.

One major limitation of this study is the possibility of recall bias about the exact onset age of the first mood episode of BPI, particular when there was a long history of the illness. Previous studies have however suggested that age at onset can be assessed reliably^61^. The preparation of life chart containing detailed clinical course and treatment based on a semi-structured clinical interview plus a thorough medical chart review for individual patients should have overcome this potential limitation satisfactorily.

In summary, we have identified a genetic locus rs11127876 in *CADM2* gene to be associated with early-onset BPI. The finding has reflected the co-sharing genetic features of psychiatric disorders and behavioral traits. Further investigations of the *CADM2* biological function in BPI are warranted.

## Supplementary information

Supplementary Materials
