# Supplementary material for: Genome-wide association study of early-onset bipolar I disorder in the Han Taiwanese population
Source: Transl Psychiatry. 2021 May 20;11:301. doi: 10.1038/s41398-021-01407-6 (PMC8137921; doi:10.1038/s41398-021-01407-6)
Supplement: Supplementary file 1 — Supplementary Materials [file 41398_2021_1407_MOESM1_ESM.docx]

**Supplementary Online Content**

**eFigure 1**: The principal component analysis plot of the 82 samples with HumanOmni1-Quad BeadChip and HumanOmni2.5-Quad BeadChip genotyping data.

**eFigure 2**: The principal component analysis plot of the 1306 GWAS samples with two chips.

**eFigure 3**: Regional plots of genomic location of *CADM2* genetic variants with genotyping data and association P values (-log_10_P).


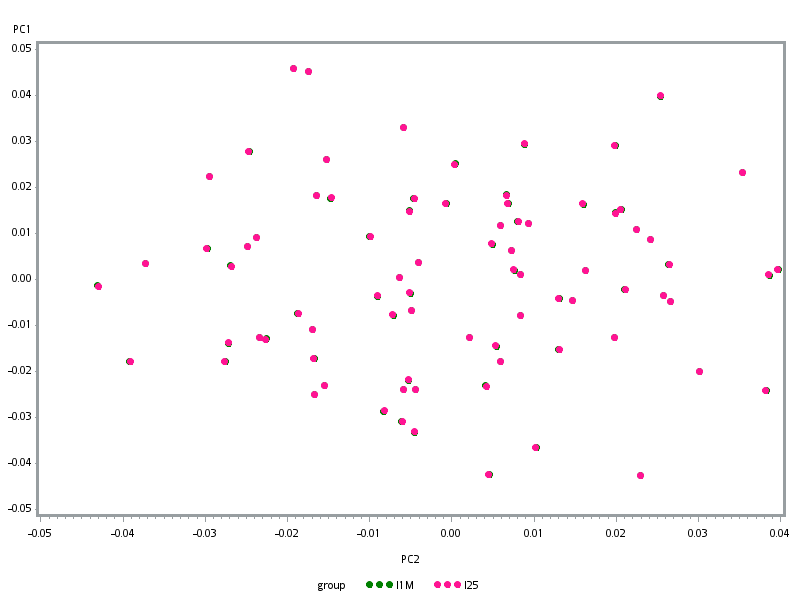


**eFigure 1. The principal component analysis plot of the 82 samples with HumanOmni1-Quad BeadChip and HumanOmni2.5-Quad BeadChip genotyping data.** The Y and X axes are the first and second dimensions from principal component analysis based on the genome-wide IBS pairwise distances among the 82 subjects. Green crosses represented HumanOmni1-Quad BeadChip and pink for HumanOmni2.5-Quad BeadChip genotyping data. The two axes correspond to a reduced representation of 10,000 randomly selected SNPs into two dimensions. The green and pink dots are almost overlapping, indicating a high consistency of the two genotyping chips.


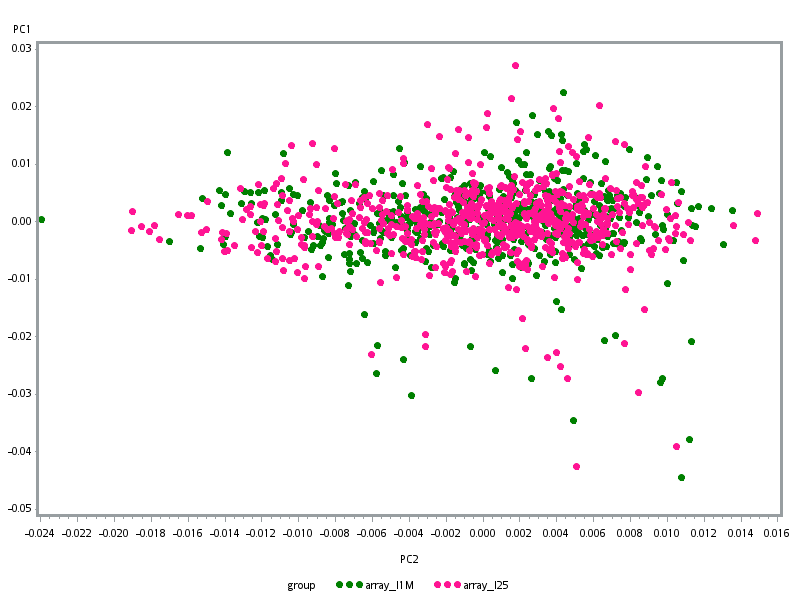


**eFigure 2 The principal component analysis plot of the 1306 GWAS samples with two chips.** The Y and X axes are the first and second dimensions from principal component analysis based on the genome-wide IBS pairwise distances among the 1306 GWAS subjects. Green crosses represented subjects with HumanOmni1-Quad BeadChip and pink for subjects with HumanOmni2.5-Quad BeadChip. The two axes correspond to a reduced representation of 10,000 randomly selected SNPs into two dimensions. No stratification nor cryptic relationship among the 1306 subjects was found.


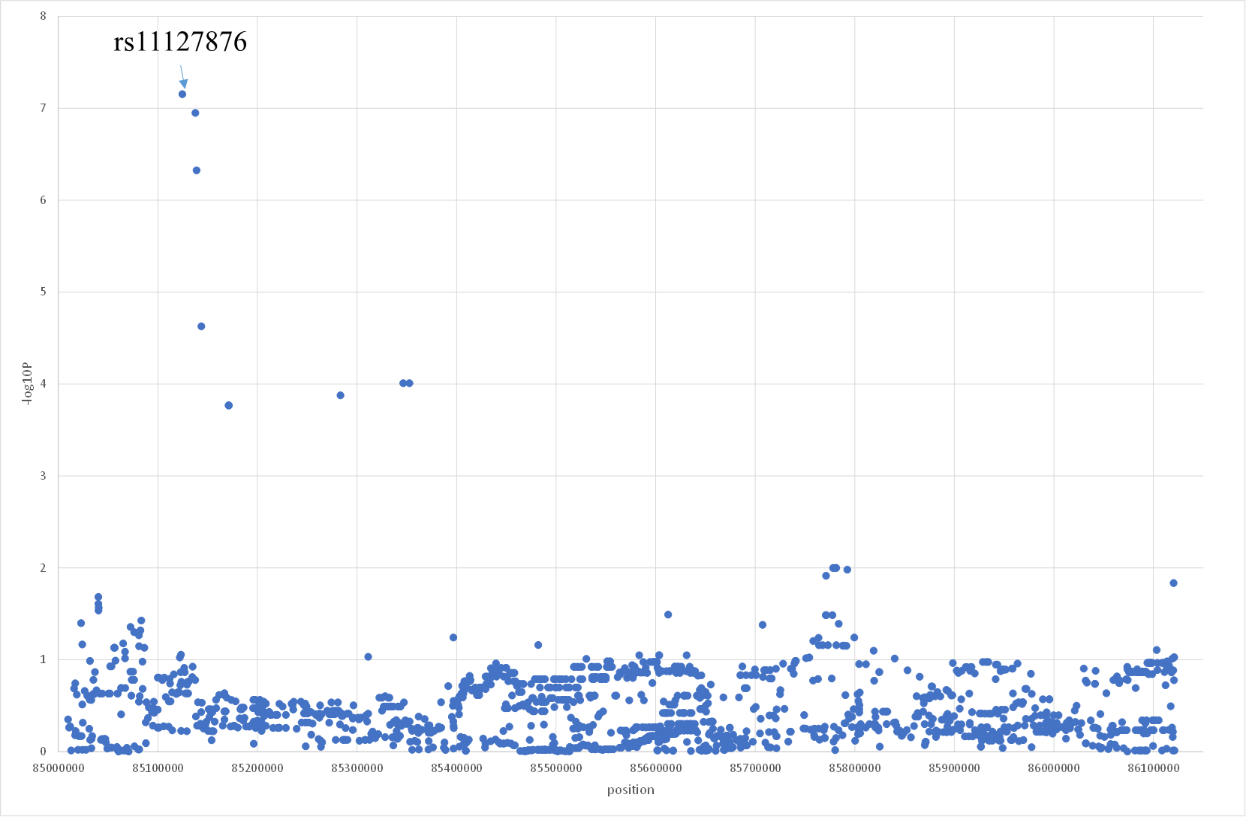


**eFigure 3. Regional plots of genomic location of *CADM2* genetic variants with genotyping data and association p values (-log_10_P)**. The SNP rs11127876 is the lead SNP (p=7.04×10^-8^) in *CADM2* genomic region. The genomic region of *CADM2* locates in chromosome 3, from 85008133 to 86123579 base pair (GRCh37.p13). Six SNPs in *CADM2* genomic region were found to show nominal statistical significance (P<0.0001), including rs11127876 (3:85124697), rs66500121 (3:85137683), rs9883252 (3:85138818), rs9814600 (3:85143683), rs1452123 (3:85346439), and rs13433706 (3:85352769).
